# Mining of chicken muscle growth genes and the function of important candidate gene *RPL3L* in muscle development

**DOI:** 10.3389/fphys.2022.1033075

**Published:** 2022-11-03

**Authors:** Shudai Lin, Mingjian Xian, Tuanhui Ren, Guodong Mo, Li Zhang, Xiquan Zhang

**Affiliations:** ^1^ College of Coastal Agricultural Sciences, Guangdong Ocean University, Zhanjiang, Guangdong, China; ^2^ Department of Animal Genetics Breeding and Reproduction, College of Animal Science, South China Agricultural University, Guangzhou, China

**Keywords:** chicken, muscle development, transcriptome, data mining, indel, ribosomal protein L3-like (RPL3L)

## Abstract

The birth weight of chickens does not significantly affect the weight at slaughter, while the different growth rate after birth was one of the important reasons for the difference in slaughter weight. Also, the increase in chickens’ postnatal skeletal muscle weight is the main cause of the slaughter weight gain, but which genes are involved in this biological process is still unclear. In this study, by integrating four transcriptome datasets containing chicken muscles at different developmental times or different chicken tissues in public databases, a total of nine candidate genes that may be related to postnatal muscle development in chickens were obtained, including *RPL3L*, *FBP2*, *ASB4*, *ASB15*, *CKMT2*, *PGAM1*, *YIPF7*, *PFKM*, and *LDHA*. One of these candidate genes is *RPL3L*, whose 42 bp insertion/deletion (indel) mutation significantly correlated with multiple carcass traits in the F2 resource population from Xinghua chickens crossing with White Recessive Rock (WRR) chickens, including live weight, carcass weight, half eviscerated weight, eviscerated weight, breast meat weight, wing weight, leg muscle shear force, and breast muscle shear force. Also, there was a very significant difference between different genotypes of the *RPL3L* 42 bp indel mutation in these trains. Further experiments showed that *RPL3L* was highly expressed in chicken skeletal muscle, and its overexpression could promote the proliferation and inhibit the differentiation of chicken myoblasts by regulating *ASB4* and *ASB15* expression. Our findings demonstrated that the *RPL3L* 42 bp indel may be one of the molecular markers of chicken weight-related traits.

## Introduction

Growth traits are one of the most important economic traits of chickens, and individual body weight is one of the most concerned economic indicators. About 40% of the body weight of a chicken comes from its skeletal muscles (Schiaffino and Reggiani, 2011). The development of skeletal muscle is closely related to meat yield and quality. Skeletal muscle composed of breast and leg muscles is one of the important protein sources for humans. Chicken skeletal muscle weight is determined by the number of muscle fibers and volume of muscle fibers, and postnatal skeletal muscle growth occurs primarily by increasing muscle fiber volume rather than muscle fiber number (Smith, 1963). The increase in muscle fiber volume is essentially the result of cellular protein accumulation, a net increase in protein synthesis minus protein degradation (Pereira et al., 2017). The rate of protein synthesis depends on two factors: translation efficiency and translation capacity, corresponding to protein synthesis per unit of RNA and the total amount of ribosomes (Figueiredo and McCarthy, 2019). Although much progress has been made in the past in exploring the molecular mechanisms underlying chicken skeletal muscle growth and development, most studies have focused on the embryonic stage (Davis et al., 2015; Taylor, 2017; Li Y. et al., 2019). It has been reported that the birth weight of chickens does not significantly affect the weight at slaughter (7–8 W) (Lara et al., 2005). However, chickens of different breeds but with similar birth weights can have large differences in the weight of 7-day-olds and 41-day-olds (Tona et al., 2017) or at slaughter (Dou et al., 2017). In other words, the growth rate of different chicken breeds may not differ much during the embryonic period. The difference in growth rate after birth is one of the important reasons for the difference in slaughter weight. However, the current research studies have not been able to fully explain this phenomenon.

The development of chicken skeletal muscle can be divided into two stages: the embryonic stage and postnatal stage. Chicken embryonic muscle grows mainly through the proliferation of myoblasts. At the late embryonic stage, muscle satellite cells attach to the lower basal layer of mature muscle fibers, and their main function is to participate in postnatal muscle damage repair and hypertrophy (Gros et al., 2005). Therefore, the number of myoblasts affects not only the number of muscle fibers but also the number of satellite cells present during postnatal growth. The development of chicken skeletal muscle during the entire embryonic period is mainly divided into the following stages: formatting the primary muscle fibers (primary fibers, also known as primary myotubes) at about 7 days of incubation, completing the secondary muscle fibers (secondary fibers, also known as the formation of secondary myotubes) at about 12 days of incubation, and basically completing the differentiation of myotubes and fixing the number of muscle fibers at about 18 days of incubation (Picard et al., 2010; Chal and Pourquie, 2017). The metabolism and contraction functions of muscle fibers mature within 3 days after birth, after which the main changes in muscle fibers are thickening and muscle fiber type switching (Smith, 1963; Chal and Pourquie, 2017). Due to the fusion of muscle satellite cells with muscle fibers during the late embryonic stage of chickens, the volume of muscle fibers increases, thereby limiting the increase in muscle weight at birth (Egner et al., 2016). After hatching, the rapidly proliferating muscle satellite cells fuse with the muscle fiber, thereby providing a large number of nuclei for the muscle fiber. More nuclei can enhance the transcription efficiency of the muscle fiber and improve the protein synthesis ability and the muscle growth ability (Dumont et al., 2015; Murach et al., 2018). It has been reported that the content of muscle satellite cells in fast-growing large broiler breeds was significantly higher than that in common breeds (Velleman et al., 2000). Although muscle satellite cells are in a resting state under normal conditions, they will be activated by the action of some specific factors. For example, muscle satellite cells will be activated when muscles are injured, and the cells will re-enter the process of proliferation and differentiation. In addition, the muscle-enhancing effect achieved by exercise often utilizes this feature of muscle satellite cells (Murach et al., 2018; Dornen and Dittmar, 2021).

Making full use of bioinformatics methods to study the regulation mechanism of poultry genetic information on phenotypic traits can effectively improve the speed of poultry breeding. Chickens with similar birth weights of different breeds can vary widely in slaughter weight. Previous research studies have not fully elucidated the phenomenon. Are there some specifically expressed genes in regulating chicken muscle growth and development after they are born? Therefore, this study aims to obtain the key genes and their signaling pathways that may affect the muscle growth and development of chickens after birth by integrating the chicken muscle-related RNA-seq data in the public database and validating one of the candidate genes, *RPL3L*, in chicken muscle growth and development. The results of this study will provide a theoretical reference for the study of chicken muscle growth and development.

## Materials and methods

### Data sources

In this study, four datasets were downloaded from three databases, including the Gene Expression Omnibus (GEO, https://www.ncbi.nlm.nih.gov/geo/), the European Nucleotide Archive (ENA, https://www.ebi.ac.uk/ena) (Harrison et al., 2021), and the National Genomics Data Center, China National Center for Bioinformation (CNCB-NGDC, https://ngdc.cncb.ac.cn/) (CNCB-NGDC Members and Partners, 2022).

The PRJCA001556 dataset in the original study (Li et al., 2020) contained transcriptome data of Shouguang chickens’ breast muscle tissues from 17 individuals at 6-time points, including the embryo day (E) 12, E17, the age of 1 day (D), 2 weeks (W), 8 W, and 14 W. In our study, the transcriptome data of 11 individuals were selected from 4-time points for analysis, including E12, E17, D1, and 8 W. The PRJCA001192 dataset in the original study (Xing et al., 2020) contained transcriptome data of Jingxin Yellow chickens’ breast muscle and abdominal adipose tissues from 27 individuals at 9-time points of E12, E17, D1, 1 W, 3 W, 8 W, 14 W, 20 W, and 26 W. In the current study, the data of breast muscle tissues from 12 individuals at 4-time points of E12, E17, D1, and 8 W were selected for analysis. In the original study (Jin et al., 2021), the GSE162148 dataset had the transcriptome data of seven tissues from 21 Tibetan chicken individuals, including skeletal muscle, liver, heart, spleen, lung, kidney, and fat. In the present study, the data from six tissues from 18 individuals were selected for analysis, including breast muscle, liver, spleen, lung, kidney, and fat. The PRJNA665193 dataset in the original study (Kern et al., 2021) contained transcriptome data of eight tissues from 16 White Leghorn chickens, including skeletal muscle, liver, spleen, lung, fat, cerebellum, cerebral cortex, and hypothalamus. All data from this dataset were selected for analysis in our study. The fastq format files of the datasets PRJCA001556 (Li et al., 2020), PRJCA001192 (Xing et al., 2020), and PRJNA665193 (Kern et al., 2021) were directly downloaded through FileZilla FTP Client (FileZilla 64-bit installation version, https://www.filezilla.cn/) and IBM Aspera Connect (ibm-aspera-connect-3.8.1.161,274-linux). The SRA file of GSE162148 (Jin et al., 2021) was downloaded through IBM Aspera Connect and used the convert SRA to fastq files plugin of TBtools (TBtools_windows-x64_1_09867, Chen et al., 2020) to convert it into a fastq format file. The information on all the datasets used in this study is listed in Supplementary Table S1.

### Quality control and gene expression quantification

Moreover, fastp (version 0.23.2, Chen et al., 2018) software was used to perform quality control on raw data, including removing adapter sequences and trimming low-quality reads. Also, Kallisto (Kallisto 0.44.0, Bray et al., 2016) software was used to perform unmatched direct quantification of quality-controlled sequencing data. The result of the Kallisto analysis was the expression level at the transcript level, which was converted into gene expression level using the tran value sum plugin of TBtools software (Kern et al., 2021).

### Principal component analysis

Principal component analysis (PCA) was performed on the data using the basic PCA analysis plugin of TBtools software (Chen et al., 2020).

### Differentially expressed gene analysis and functional enrichment analysis

Differentially expressed gene (DEG) analysis was performed on the read count obtained by Kallisto using the differential gene expression analysis (DESeq2, Love et al., 2014) plugin of TBtools software (Chen et al., 2020), and the upregulated genes in each group were screened with log2|FC| > 1 and FDR < 0.05 as thresholds. Gene Ontology (GO) and Kyoto Encyclopedia of Genes and Genomes (KEGG) enrichment analysis of DEGs were performed *via* the website https://biit.cs.ut.ee/gprofiler/gost. The GO database classified DEGs into three categories according to their molecular functions (MF), cellular components (CC), and biological processes (BP) involved.

### Protein–protein interaction network analysis

The protein–protein interaction (PPI) network was constructed using the Search Tool for the Retrieval of Interacting Genes (STRING) (version 10.0, https://cn.string-db.org/) online database. Cytoscape (version 3.6.1, https://cytoscape.org/) was used to visualize the PPI network, and its cytoHubba plugin was used to screen the top 10 as hub genes (key genes) according to the degrees of nodes.

### Insertion/deletion (indel) typing

The genomic DNA samples of 350 individuals’ blood from the F2 resource population of Xinghua chickens crossed with WRR chickens were extracted following the instructions of the HiPure Blood DNA Mini Kit (Guangzhou Magen Biotechnology Co., Ltd., Guangzhou, China). All of them were amplified by PCR with RPL3L-INDEL primers (Supplementary Table S2), and the products were analyzed by electrophoresis on agarose gel at a concentration of 2%.

### Animals and cells

All animal experiments performed in this study were approved by the Institutional Animal Protection and Utilization Committee of South China Agricultural University (approval ID: SCAU#0014). Furthermore, the care and use of animals complied with the local animal welfare laws, guidelines, and policies. The chicken embryos used in this study were as previously described (Luo et al., 2014). The experimental animals were purchased from Zhaoqing Fengkai Zhicheng Poultry Breeding Co., Ltd. During hatch, the temperature was controlled at 38.5°C, 38°C, 37.9°C, and 37.3°C–37.5°C in the first 6 days, 7–14 days, 15th day, and 16–21 days of hatching, respectively. The relative humidity was maintained at 60%–70%, 50%–55%, and 65%–70% for the 1–7 days, 8–16 days, and after the 17th day of incubation, respectively. The chicks after hatching drank freely and were fed with chick feed which was provided by Zhaoqing Fengkai Zhicheng Poultry Breeding Co., Ltd. The breast muscle samples were collected from embryonic day 10 (E10) to E15 and post-hatch day 1 chicks, and the breast muscle, leg muscle, heart, liver, spleen, lung, brain, cerebellum, pituitary, abdominal fat, and cartilage samples were collected from 7-week-old Xinghua chickens. The information of 100 individuals from 15 chicken populations were as previously described (Luo et al., 2020). The 15 chicken populations were composed of nine Chinese nationwide indigenous chicken breeds, including six Huiyang Bearded chickens (BC), 20 Hetian chickens (HT), six Baier Yellow chickens (BEH), six Xianju chickens (XJ), six Liyang chickens (LY), six Jining Bairi chickens (BR), six Yunyang Da chickens (YY), six Lindian chickens (LD), and six Tulufan gamecock chickens (TLF); four typical commercial populations, including six White Leghorn chickens (LH), six White Recessive Rocks (WRR), six Cobb RS308 chickens (RS), and six Rhode Island Reds (RIR); one Red jungle fowl population from Guangxi Province (five individuals, RJF) and one gamecock population from Laos (three individuals, Laos).

The primary myoblasts of chicken were isolated from the leg muscles of E11 chicks and cultured in DMEM (Invitrogen Trading Co. Ltd., Shanghai, China) as previously characterized (Luo et al., 2014). Myoblast differentiation and DF-1 cell culturing were as previously reported (Luo et al., 2015). The chicken satellite cells were isolated from the breast muscle of 5-day-old Xinghua chicks and cultured in DMEM/F12 (Invitrogen) were the same as previously described (Han et al., 2019).

### Plasmid construction and cell transfection

The overexpression vector of the chicken *RPL3L* gene coding sequence (CDS) region (pcDNA3.1-RPL3L) and *RPL3L* gene indel firefly luciferase reporters (PGL3-RPL3L-II and PGL3-RPL3L-DD) were synthesized by Wuhan GeneCreate Biological Engineering Co., Ltd. The cell transfection was performed using Lipofectamine^TM^ 3000 Transfection Reagent following the manufacturer’s instructions (Invitrogen).

### Quantitative real-time PCR

Total RNA of breast muscle tissues of Xinghua chickens before or after birth, different tissues of 7 W Xinghua chickens, and the primary myoblasts and satellite cells of chicken that transfected with or without overexpression of RPL3L was extracted according to the instructions of the HiPure Total RNA Mini Kit (Guangzhou Magen Biotechnology Co., Ltd., Guangzhou, China). Then, following the instructions of the MonScript™ RTIII All-in-One Mix with dsDNase kit (Monad Biotech Co. Ltd., Wuhan, China) to synthesize cDNA. Quantitative real-time PCR (qRT-PCR) was carried out according to the instructions of the MonAmp™ ChemoHS qPCR Mix kit (Monad Biotech Co. Ltd., Wuhan, China). Fluorescence quantitative results were calculated by Excel software to calculate the 2^-△△Ct^ value to compare the relative gene (*β-actin*) expression. All primers used for qRT-PCR were listed in Supplementary Table S2.

### Cell proliferation assay

A total of 3 × 10^3^ primary myoblasts of chicken per well were seeded in 96-well plates, after 0, 6, 12, 24, and 48 h transfection with pcDNA3.1-RPL3L or pcDNA3.1, the Cell Counting Kit-8 (CCK-8) experiment was performed according to the instructions of the CCK-8 kit (Beyotime Biotechnology Co., Ltd., Shanghai, China). A total of 5 × 10^5^ primary myoblasts of chicken per well were seeded in 12-well plates, after 48 h transfection with pcDNA3.1-RPL3L or pcDNA3.1, the cell cycle assay was performed as previously described (Luo et al., 2015), and the EdU experimental was carried out according to the protocol of the EdU Cell Proliferation Detection kit (Ribobio Co., Ltd., Guangzhou, China). All experiments were repeated at least three times.

### Western blotting

A total of 1.2 × 10^6^ primary myoblasts of chicken per well were seeded into 6-well plates, cultured in DMEM, and transfected with pcDNA3.1-RPL3L or pcDNA3.1 plasmids. After 48 h of transfection, the whole-cell lysate was extracted by the lysate (a 10:1 ratio of RIPA lysate and PMSF) and their concentration was determined using a bicinchoninic acid (BCA) protein assay kit (Beyotime Biotechnology Co., Ltd., Shanghai, China). A total of 5 µg proteins were separated by 8%–12% SDS-PAGE and transferred onto a polyvinylidene difluoride (PVDF) membrane (Millipore, Billerica, MA, USA) that was pre-soaked in methanol. The membrane was then blocked with Quick Blocking Buffer (Beyotime Biotechnology Co., Ltd., Shanghai, China) for 15 min at room temperature before incubation overnight at 4°C with primary detection antibodies, including the primary antibodies specific for anti-RPL3L (Abcepta Biotech Ltd., Co., Suzhou, China; 1:500), anti-Myhc, anti-Myog, anti-Myod (Thermo Fisher Scientific; 1:500 for each of them), and anti-β-actin (Beyotime Biotechnology Co., Ltd., Shanghai, China; 1:1000). The PVDF membrane was washed three times with western wash buffer (Beyotime Biotechnology Co., Ltd., Shanghai, China), followed by incubation for 2 h at room temperature with the secondary antibody, horseradish peroxidase (HRP)-labeled anti-mouse and anti-rabbit immunoglobulin G (IgG) (Beyotime Biotechnology Co., Ltd., Shanghai, China; 1:2000). Bands were detected using the BeyoECL Star chemiluminescence substrate (Beyotime Biotechnology Co., Ltd., Shanghai, China).

### Dual-luciferase reporter assay

A total of 5 × 10^4^ DF-1 cells/well were seeded in 96-well plates and transfected with a 1:20 ratio of PGL3-RPL3L-II or PGL3-RPL3L-DD to renella luciferase reporter (pRL-TK) plasmid. After 24 h of transfection, the cells were lysed and centrifuged at 12,000 rpm for 1 min to collect the supernatant. The dual-luciferase reporter assay was performed according to the instructions of the Dual-Glo^®^ Luciferase Assay System (Promega Biotech Co., Ltd., Beijing, China).

### Transcription factor prediction

The online website http://gene-regulation.com/pub/programs/alibaba2/index.html was used to predict the transcription factor.

### Association analysis and statistical analysis

As previously described (Li W. et al., 2019; Lin et al., 2021; Wei et al., 2022), the mixed linear model in SPSS software (version 24.0) was used to analyze the association between different genotypes of indel and the different phenotypic traits of F2 resource population from Xinghua chickens crossing with WRR chickens. The analysis model was as follows,

$$Y_{ijklm}=\mu +G_{i}+S_{j}+H_{k}+f_{l}+e_{ijklm}.$$

Among them, Y_ijklm_ represents the trait phenotype value of the individual, μ represents the overall population mean, G_i_ is the fixed effect of the marker genotype (*i* = 3), S_j_ represents the fixed effect of sex, H_k_ represents the fixed effect of the batch, f_l_ is the fixed effect of the family, and e_ijklm_ represents the random error. A least-squares analysis was used to investigate the effect of the polymorphic genotypes on the target traits. *p* < 0.05 indicated a significant level, and Bonferroni’s adjustment was performed to control for multiple comparisons.

Data from the other experiments were shown as the mean values with the standard error of the mean. Statistical differences were determined using Student’s t-test or one-way ANOVA. *p* < 0.05 was considered to indicate statistical significance.

## Results

### Results of quality control and principal component analysis

After filtering the PRJCA001556 dataset, the average size of single-sample sequencing data was 11.4 Gb, the average number of reads was 76.36 M, and the Q20 and Q30 were 98.12% and 94.64%, respectively (Supplementary Table S3). From the filtered results of the PRJCA001192 dataset, the average size of single-sample sequencing data was 7.45 Gb, and the average number of reads was 60.93 M. Q20 and Q30 were 95.16% and 89.83%, respectively (Supplementary Table S3). After filtering the GSE162148 dataset, the average size of single-sample sequencing data was 14.26 Gb, and the average number of reads was 95.46 M. Q20 and Q30 were 97.78% and 94.06%, respectively (Supplementary Table S3). As a result of the filtered PRJNA665193 dataset, the average size of single-sample sequencing data was 13.00 Gb, the average number of reads was 132.90 M, and the Q20 and Q30 rates were 98.80% and 96.12%, respectively (Supplementary Table S3). These results showed that the overall quality of the clean reads of the sequencing data fulfilled the requirements of subsequent data analysis.

**FIGURE 1:**
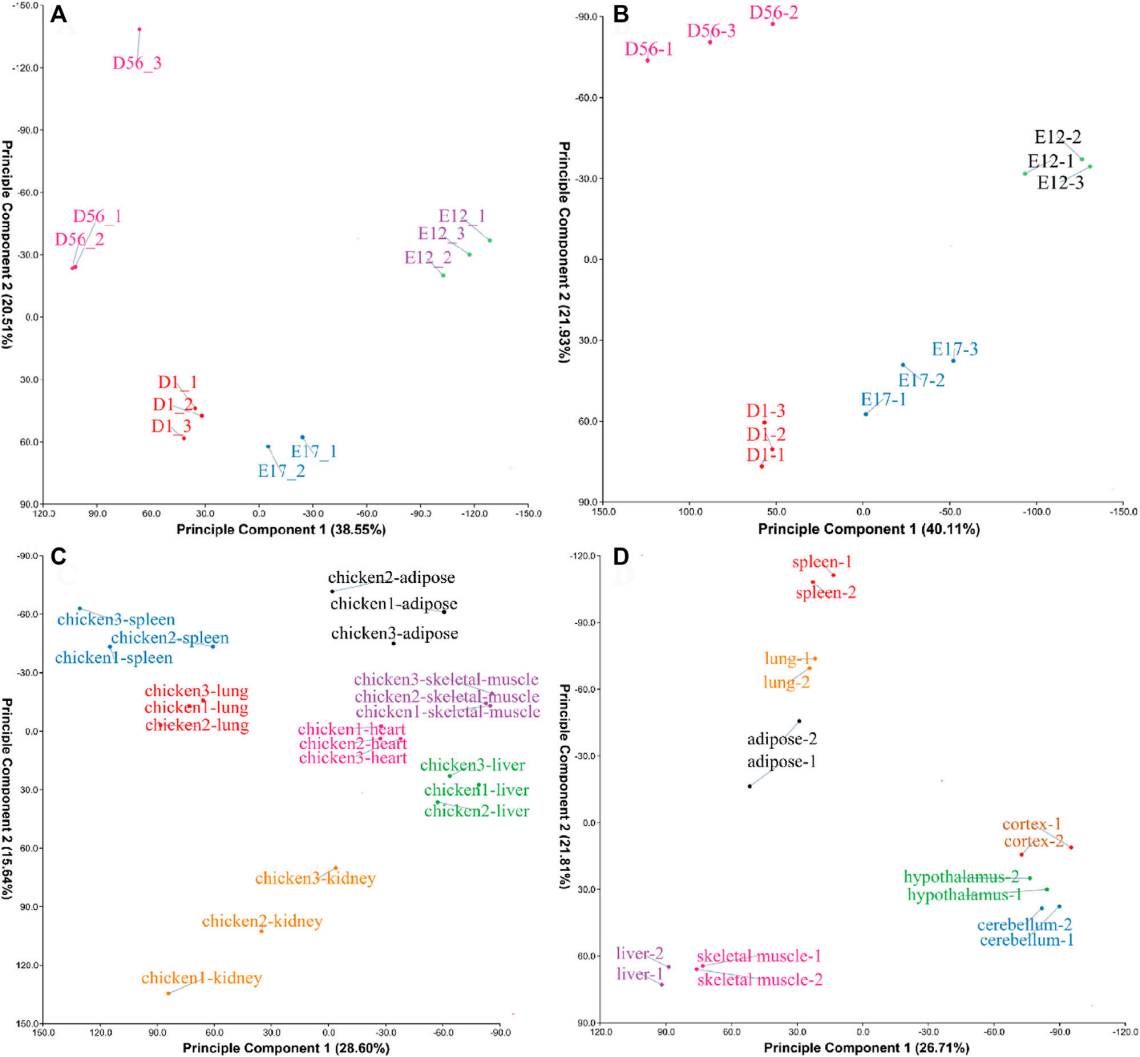
Principal component analysis (PCA) of samples from different datasets. **(A)** The PCA result of the PRJCA001556 dataset; **(B)** PCA result of the PRJCA001192 dataset; **(C)** PCA result of the GSE162148 dataset; and **(D)** PCA result of the PRJNA665193 dataset.

The four datasets PRJCA001556, PRJCA001192, GSE162148, and PRJNA665193 were found to be clustered together after performing PCA (Figure 1), suggesting that the samples were well grouped and could be used for further analysis.

### Differentially expressed genes and functional enrichment analysis

**FIGURE 2:**
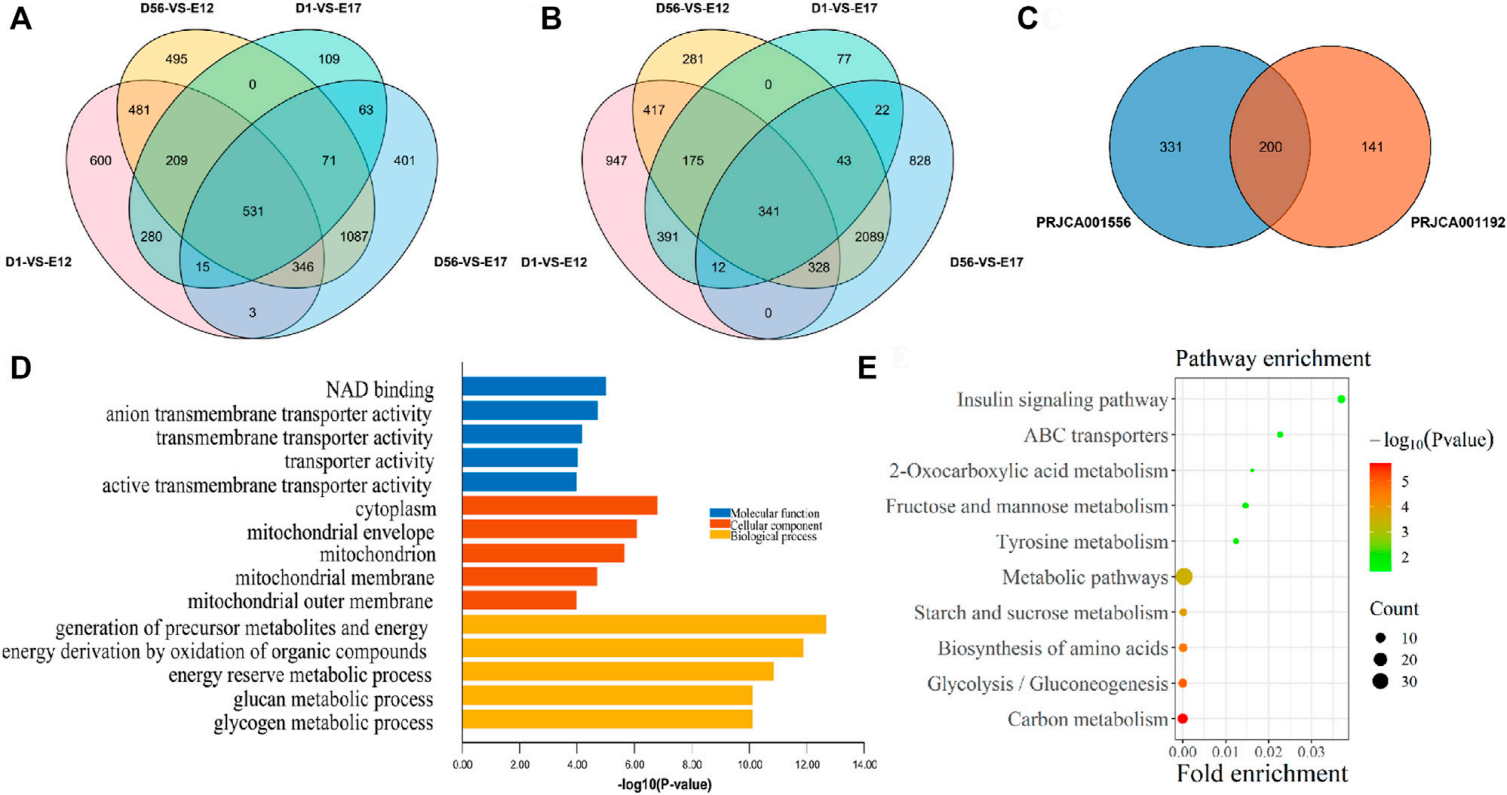
Functional enrichment analysis of postnatal-specific highly expressed genes. **(A)** Venn diagram of DEGs from the PRJCA001556 dataset; **(B)** Venn diagram of DEGs from the PRJCA001192 dataset; **(C)** overlapping DEGs between PRJCA001556 and PRJCA001192 datasets; **(D)** GO enrichment analysis results of postnatal-specific highly expressed genes; and **(E)** KEGG enrichment analysis results of postnatal-specific highly expressed genes.

In order to screen the genes that are specifically highly expressed in chickens after birth, the D1, 8 W muscle transcriptome data were compared with those of E12 and E17 from the PRJCA001556 and PRJCA001192 datasets, respectively. As a result, compared with E12 and E17, a total of 531 DEGs were upregulated in both D1 and 8 W in the PRJCA001556 dataset (Figure 2A), and 341 differentially expressed genes (DEGs) were upregulated in both D1 and 8 W in the PRJCA001192 dataset (Figure 2B). Additionally, a total of 200 postnatal upregulated DEGs were overlapped in these two datasets (Figure 2C). According to the top five GO terms with the smallest *p*-values in the three categories of MF, CC, and BP of the 200 DEGs between the embryonic stage and after hatching, the GO enrichment results of postnatal-specific highly expressed genes included NAD binding, anion transmembrane transporter activity, transmembrane transporter activity, transporter activity, and active transmembrane transporter activity (Figure 2D). As a result of KEGG pathway enrichment analysis, the significantly enriched pathways included the insulin signaling pathway, ABC transporter, and 2-oxocarboxylate metabolism(Figure 2E).

**FIGURE 3:**
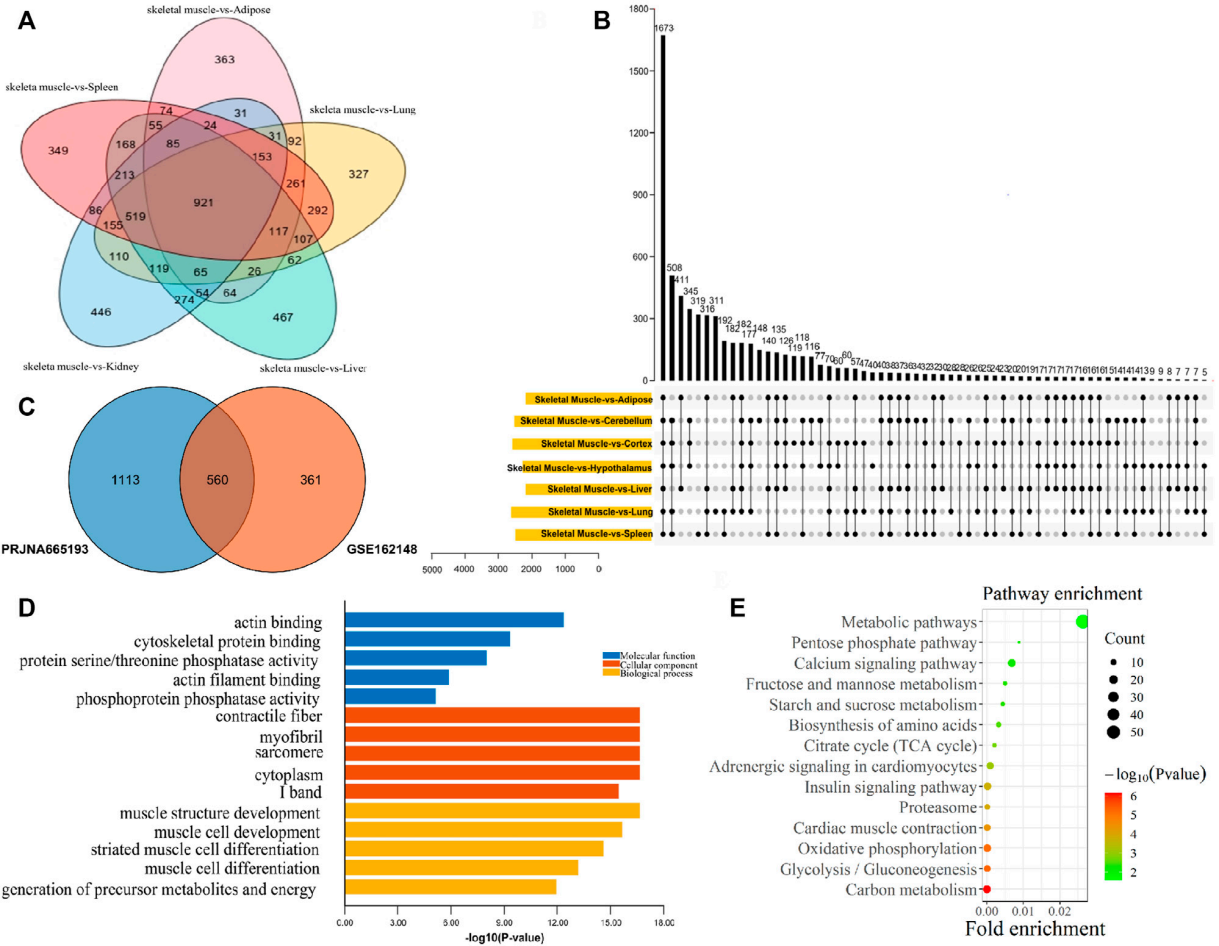
Functional enrichment analysis of muscle-specific highly expressed genes. **(A)** Venn diagram of DEGs from the GSE162148 dataset; **(B)** Venn diagram of DEGs from the PRJNA665193 dataset; **(C)** overlapping DEGs between the postnatal-specific high-expressed gene set and the muscle-specific high-expressed gene set; **(D)** GO enrichment analysis results of muscle-specific highly expressed genes; and **(E)** KEGG enrichment analysis results of muscle-specific highly expressed genes.

For exploring genes that were specifically highly expressed in muscle, the muscle tissues and non-muscle tissues transcriptome data of GSE162148 and PRJNA665193 datasets were analyzed, respectively. As a result, compared with non-muscle tissues, there were 921 upregulated DEGs in muscle tissues in the GSE162148 dataset (Figure 3A), and 1673 upregulated DEGs in muscle tissue in the PRJNA665193 dataset (Figure 3B). In addition, a total of 560 muscle upregulated DEGs were overlapped in these two datasets (Figure 3C). According to the top five GO terms with the smallest *p*-values in the three categories of MF, CC, and BP of the 560 DEGs upregulated in muscle tissues, the GO enrichment results of muscle-specific highly expressed genes included actin binding, cytoskeletal protein binding, and protein serine/threonine phosphatase activity (Figure 3D). As muscle-specific were highly expressed genes, they were significantly enriched in various GO terms related to muscle composition and muscle development. Also, the result of the KEGG pathway enrichment analysis found that they were significantly enriched in the pathways of carbon metabolism, glycolysis/gluconeogenesis, oxidative phosphorylation, and myocardial contraction (Figure 3E).

**FIGURE 4:**
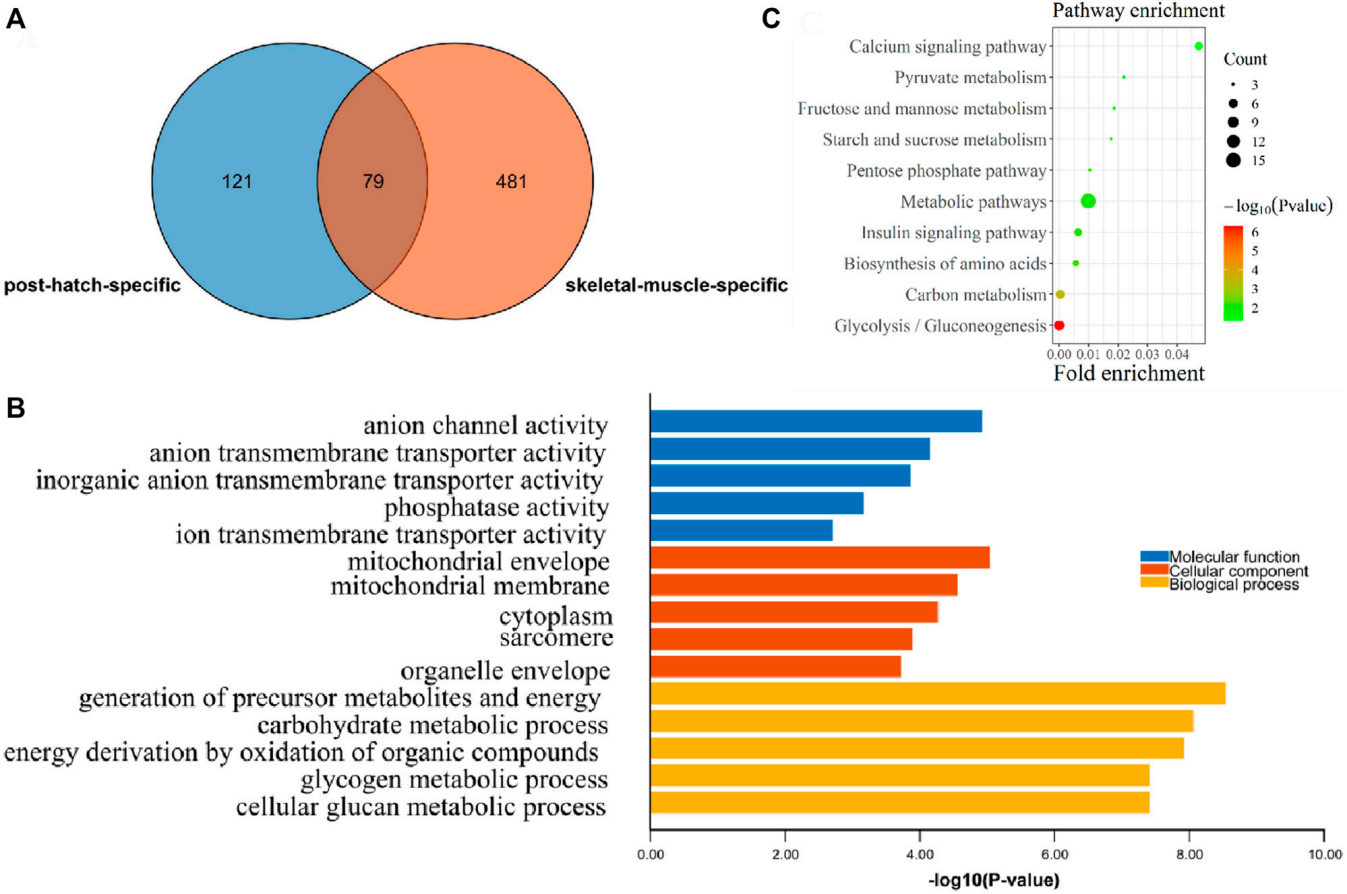
Functional enrichment analysis of postnatal muscle-specific highly expressed genes. **(A)** Overlapping DEGs between the postnatal-specific high-expressed gene set and the muscle-specific high-expressed gene set; **(B)** GO enrichment analysis results of postnatal muscle-specific highly expressed genes; and **(C)** KEGG enrichment analysis results of postnatal muscle-specific highly expressed genes.

In order to find the postnatal and muscle-specific high-expression genes, the postnatal-specific high-expression gene set and the muscle-specific high-expression gene set obtained earlier were intersected. A total of 79 postnatal and muscle-specific highly expressed genes were screened (Figure 4A and Supplementary Table S4). From the results of GO and KEGG enrichment analysis on these 79 genes, it was found that the GO MF enrichment mainly included anion channel activity and anion transmembrane transporter activity (Figure 4B). In addition, the GO CC enrichment mainly included the mitochondrial envelope, mitochondrial membrane, cytoplasm, sarcomere, and organelle envelope, and the GO BP enrichment mainly contained the production of precursor metabolites and energy, the process of carbohydrate metabolism, and the oxidation of organic compounds to generate energy (Figure 4B). Moreover, as a result of KEGG pathway enrichment, they were significantly enriched in the pathways of glycolysis/gluconeogenesis, carbon metabolism, and amino acid biosynthesis (Figure 4C).

### Protein–protein interaction network analysis results

**FIGURE 5:**
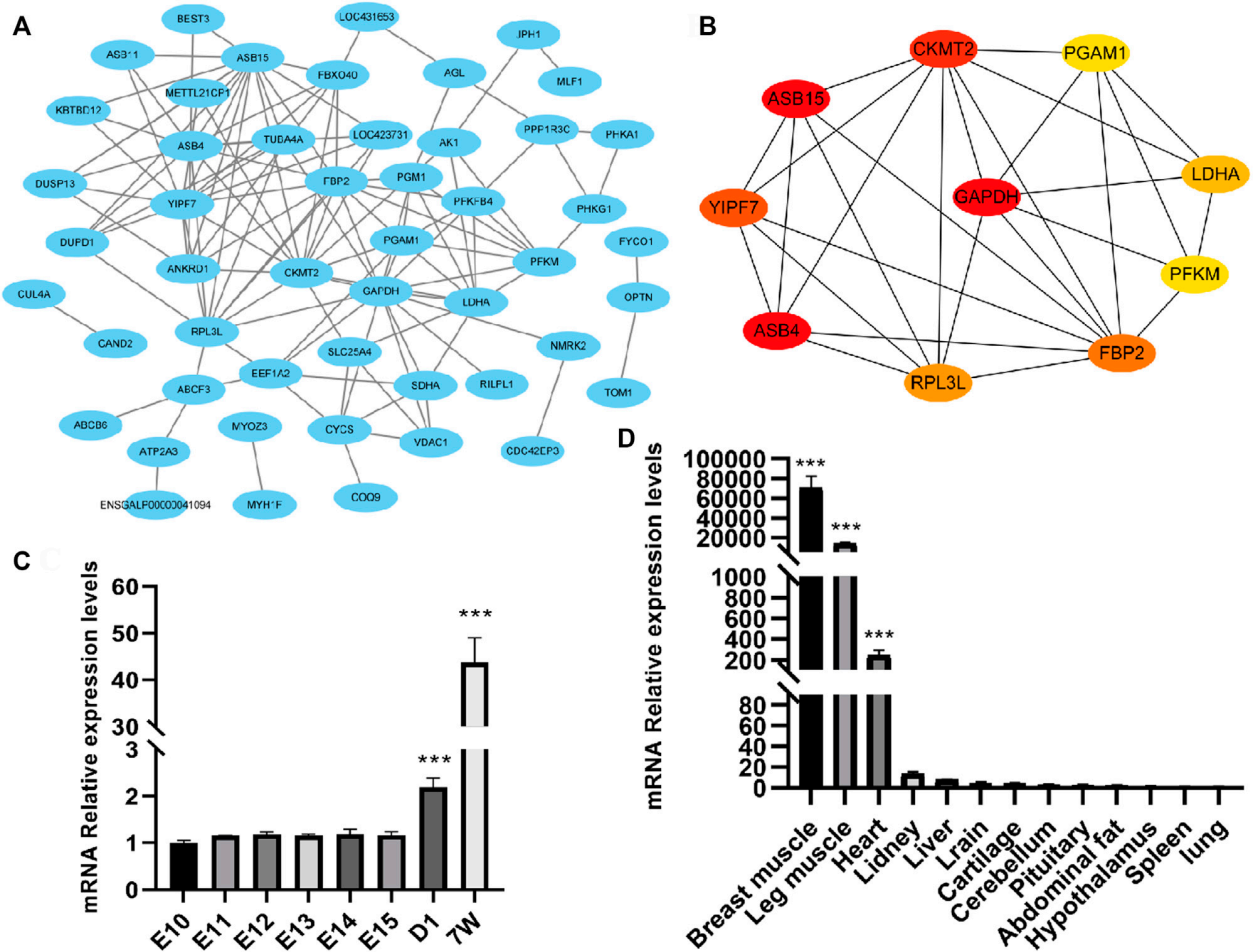
Protein–protein interaction (PPI) network analysis and validation of one of the hub genes *RPL3L*. **(A)** PPI network composed of differential genes; **(B)** top 10 hub genes according to the degree score; **(C)** expressed level of *RPL3L* in different developmental stages of the breast muscle of Xinghua chicken; and **(D)** expression level of *RPL3L* in different tissues of Xinghua chicken. ***p < 0.001.

To reveal the interactions between the 79 DEGs, we analyzed and constructed a PPI network, and screened the hub genes. As a result, there was a protein–protein interaction (PPI) network, which consisted of 50 nodes and 123 edges in total (Figure 5A). Also, 10 hub genes were screened out, including *RPL3L*, *FBP2*, *ASB4*, *ASB15*, *CKMT2*, *PGAM1*, *YIPF7*, *PFKM*, *LDHA*, and *GAPDH* (Figure 5B). Among them, except for *YIPF7* and *GAPDH,* they all have been reported to play a certain regulatory role in muscle growth and development.

Considering that the *RPL3L* has not been reported in livestock and poultry and its fold change (after birth/embryonic stage) was higher than that of the other genes, we chose *RPL3L* from the 10 hub genes for further study. From the results of qRT-PCR, it was shown that the expression level of *RPL3L* was significantly higher after birth than the embryonic stage in breast muscle tissues of Xinghua chickens (Figure 5C), and its expression level in muscle tissues of 7-week-old Xinghua chickens was also significantly higher than that in non-muscle tissues, among which breast muscle had the highest expression level (Figure 5D).

### *RPL3L* promotes chicken myoblast proliferation and inhibits myoblast differentiation

In order to preliminarily study the effect of *RPL3L* on chicken muscle development, we detected its expression level in different proliferation and differentiation stages of primary myoblasts and muscle satellite cells. It was found that *RPL3L* first decreased and then increased during the proliferation and differentiation stage of myoblasts (Supplementary Figure S1A), while in muscle satellite cells, it continued to rise along with the cell differentiation (Supplementary Figure S1B). These results suggest that *RPL3L* may be involved in the regulation of muscle growth and development.

**FIGURE 6:**
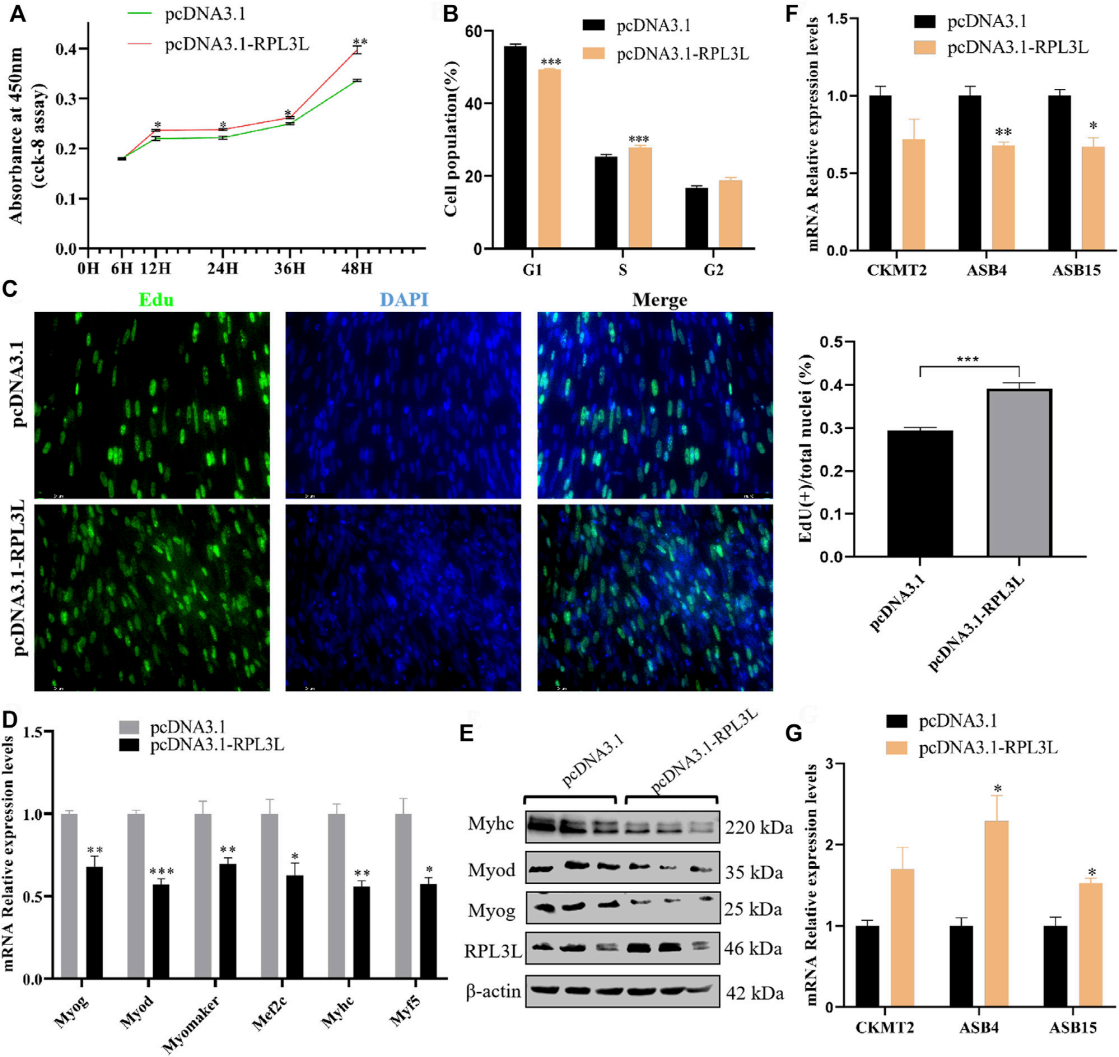
*RPL3L* promotes cell proliferation and inhibits cell differentiation of chicken primary myoblast. **(A)** CCK-8 result with the overexpressed chicken *RPL3L* and its negative control; **(B)** cell cycle assay result after transfecting with the overexpressed chicken *RPL3L* and its negative control; **(C)** EdU result with the overexpressed chicken *RPL3L* and its negative control; **(D)** mRNA expression levels of differentiation-related marker genes after overexpressing chicken *RPL3L*; **(E)** protein expression levels of differentiation-related marker genes after overexpressing chicken *RPL3L*; **(F)** expression levels of *CKMT2*, *ASB4*, and *ASB15* detected by overexpression of *RPL3L* in myoblast proliferation stage, respectively; and **(G)** expression levels of *CKMT2*, *ASB4*, and *ASB15* detected by the overexpression of *RPL3L* in the myoblast differentiation stage, respectively. ns, not significant, *p < 0.05, **p < 0.01, and ***p < 0.001.

Then, the overexpression vector of *RPL3L* was transfected into chicken primary myoblasts. The CCK-8 results showed that overexpression of *RPL3L* could significantly enhance cell viability (Figure 6A). From the result of the cell cycle assay, it was found that cells in the G1 phase were significantly reduced, and cells in the S phase were significantly increased after overexpression of *RPL3L* compared with the control group (Figure 6B). The EDU results also showed that overexpressing *RPL3L* could promote the proliferation of chicken primary myoblasts (Figure 6C). These results suggest that *RPL3L* can significantly promote the proliferation of primary myoblasts. Moreover, overexpression of *RPL3L* in the differentiation stage of myoblasts significantly inhibited the transcription levels of differentiation-related marker genes, including *Myog*, *Myod*, *Myomaker*, *Myhc*, *Mef2c*, and *Myf5* (Figure 6D), and the protein levels of *Myhc*, *Myog*, *Myod*, and *Myomaker* (Figure 6E), implying that RPL3L may inhibit primary myoblast differentiation by downregulating the expression of these differentiation-related proteins.

According to the previously mentioned PPI results, there was a certain interaction between *RPL3L* and several genes known to affect the growth and development of chicken skeletal muscle, such as *CKMT2*, *ASB4*, and *ASB15*. In this study, *RPL3L* was overexpressed in the primary myoblast proliferation phase (Supplementary Figure S1A), and it was found that *RPL3L* could inhibit the transcription of *ASB4* and *ASB15* genes in the cell proliferation phase (Figure 6F). Overexpression of *RPL3L* during differentiation could significantly promote the transcription levels of these two genes (Figure 6G). This result demonstrated that chicken *RPL3L* may regulate muscle growth and development by affecting the expression of *ASB4* or *ASB15*.

### *RPL3L* polymorphism affects chicken growth and development

From the *RPL3L* gene structure and conservation among different species, we found that the chicken *RPL3L* gene was in the length of 7359 bp, located on chicken chromosome 14, and consists of 10 exons and nine introns. The closest distance between chicken *RPL3L* and duck was found by constructing nucleotide evolutionary trees of 10 different species (data not shown). Comparing the conservation of the protein encoded by *RPL3L* in 10 different animals, including chicken, human, pig, and mouse, it was found that the RPL3L protein was very conserved, indicating that the function of RPL3L may be similar among animals (data not shown).

**FIGURE 7:**
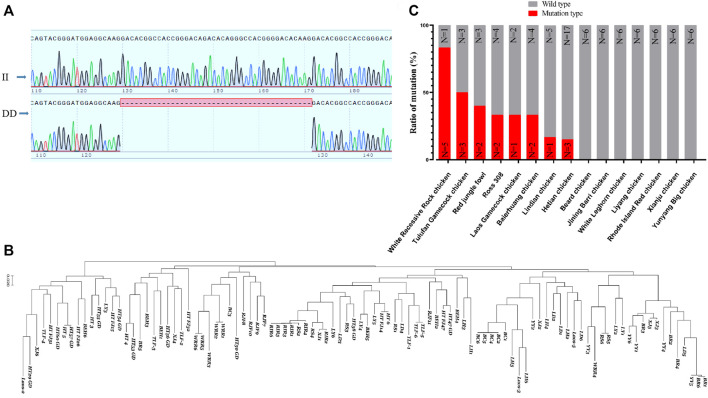
Polymorphism of Chicken *RPL3L*. **(A)** The 42 bp indel of chicken *RPL3L*; **(B)** evolutionary tree diagram of *RPL3L* in different chicken breeds; **(C)** mutation ratio of *RPL3L* 42 bp indel in different chicken breeds. BC, Huiyang Bearded Chicken; WRR, White Recessive Rock Chicken; HT, Hetian chicken; BEH, Baier Yellow chicken; XJ, Xianju chicken; LY, Liyang chicken; BR, Jining Bairi chicken; YY, Yunyang Da chicken; LD, Lindian chicken; TLF, Tulufan gamecock chicken; LH, White Leghorn chicken; RS, Cobb RS308 chicken; RIR, Rhode Island Reds; RJF, Red jungle fowl chicken; and Laos, one gamecock population from Laos.

Based on a 42 bp indel (Figure 7A) and the SNP sites of the *RPL3L* gene, an evolutionary tree was constructed. It was found that the *RPL3L* gene was strongly selected in Huiyang Bearded chicken, Rhode Island Reds chicken, and WRR chicken (Figure 7B). Also, the frequency of this 42 bp mutation in fast-growing chicken breeds such as WRR chickens was significantly higher than that in other Chinese landraces with slow-growing breeds (Figure 7C). These results imply that the mutation may be one of the important molecular markers of chicken growth traits.

Using gel electrophoresis, we classified the indels into insertion/insertion (II) type, insertion/deletion (ID) type, and deletion/deletion (DD) type (Supplementary Figure S2). The SPSS software was used to analyze the association between the indel and the growth traits of the F2 resource population from Xinghua chickens crossing with WRR chickens. It was shown that the different genotypes of this mutation were significantly correlated with multiple growth traits of chickens, including live weight, carcass weight, half eviscerated weight, eviscerated weight, breast meat weight, wing weight, leg muscle shearing force, and chest muscle shear force (*p* < 0.05) (Table 1). The results indicated that individuals with the DD genotype had significant advantages in multiple economic traits.

**TABLE 1:** Traits significantly associated with chicken *RPL3L* 42 bp indel.

Traits	Mean ± SE			*p* value
	II (*N* = 109)	ID (*N* = 158)	DD (*N* = 37)	
Live weight before slaughter (kg)	1.459 ± 0.025^a^	1.530 ± 0.022^b^	1.667 ± 0.068^b^	0.001
Carcass weight (g)	1305.028 ± 23.509	1346.920 ± 20.001	1460.879 ± 69.149	0.040
Half eviscerated weight (g)	1188.884 ± 21.813	1229.973 ± 18.866	1323.724 ± 60.750	0.031
Eviscerated weight (g)	1030.748 ± 19.284^a^	1067.442 ± 16.734^ab^	1163.635 ± 53.084^b^	0.017
Breast meat weight (g)	88.486 ± 2.167^a^	91.761 ± 1.962^ab^	102.790 ± 5.047^b^	0.008
Wing weight (g)	63.182 ± 1.178	65.275 ± 1.022	70.315 ± 3.222	0.035
Shear force of leg muscle (N)	72.838 ± 2.630	77.338 ± 2.125	62.955 ± 8.975	0.013
Shear force of breast muscle (N)	32.973 ± 1.163	30.241 ± 0.999	36.096 ± 3.351	0.046

Mean ± SE, mean ± standard error; II, indel type; ID, insertion/deletion type; DD, deletion/deletion type; N, number of individuals; different lowercase letters (a and b) in the same shoulders indicate significant differences (*p* < 0.05); with a same lowercase letter (a and ab, ab and b), and no letter mark indicates no significant difference (*p* > 0.05). The *p* values represent thesignificance between indels and traits.

### Indels of *RPL3L* influence its transcriptional activity

In order to analyze the effect of this 42 bp indel on the expression level of the *RPL3L* gene, the PGL3-RPL3L-II and PGL3-RPL3L-DD vectors were transfected into DF-1 cells for dual-luciferase reporter experiments to detect the transcriptional activity of these two genotypes of the *RPL3L* promoter. It was found that the transcriptional activity of the II genotype was significantly higher than that of the DD genotype (*p* < 0.0001) (Supplementary Figure S3A), suggesting that the 42 bp deletion could inhibit the transcriptional activity of *RPL3L*. Transcription factor prediction through online tools found that genotype DD may lose binding sites for a total of eight transcription factors, including CPE-bind, C/EBP, c-Myc, Max1, Sp1, AP-2alph, NF-1, and GATA-1 (Supplementary Figure S3B,C.

## Discussion

With the continuous development of sequencing technology, bioinformatics has played a pivotal role in the field of poultry breeding. Different biological information databases contain a large amount of poultry genetic information, and there could be new valuable information by integrated analysis of these public data again. In this study, by integrating the transcriptome sequencing data of different chicken tissues and chicken muscles at different developmental times, it was found that the GO BP terms of the differentially highly expressed genes after birth were mainly the production and metabolism of organic matter and energy. The KEGG enrichment results mainly included pathways of amino acid biosynthesis and carbon metabolism. Just like in previous studies, muscle growth and development are often accompanied by the production and metabolism of a large amount of organic matter. Skeletal muscle can absorb and utilize glucose and fatty acids, and a lack of energy can trigger muscle atrophy. Protein synthesis consumes energy, and a key sensor of cellular energy levels (AMPK) is sensitive to the nutritional status of the body (Kimura et al., 2003). The protein synthesis of skeletal muscle decreases when the body lacks energy and nutrients (Zhu et al., 2004).

From the result of the PPI network analysis of these 79 DEGs, it was found that *PFKM*, *LDHA*, *PGAM1*, *FBP2*, *CKMT2*, *ASB4*, *ASB15*, and *RPL3L* were the hug genes, which were reported to play a certain regulatory role in muscle growth and development. For example, muscle phosphofructo-1 kinase (*PFKM*) is a key regulatory enzyme of glycolysis (Garcia et al., 2009), and regulates the insulin-stimulated glycolytic pathway in chicken skeletal muscle (Seki et al., 2006). Lactate dehydrogenase A (*LDHA*) inhibits the proliferation and promotes differentiation of chicken primary myoblasts through being targeted by miR-29b-1-5p (Li et al., 2022). Studies have pointed out that white streaks and lignified chicken breasts in broiler breasts may be related to *LDHA*, but no causal relationship has been established (Malila et al., 2019). Phosphoglycerate mutase 1 (*PGAM1*) may be a potential molecular marker of chicken tenderness, and its expression is positively correlated with broiler breast muscle shear stress (Kubota et al., 2021). Fructose bisphosphatase 2 (*FBP2*) can promote fetal birth weight, and is positively correlated with the development of middle and late skeletal muscle in sheep (Xu et al., 2014). In addition, it can increase glucose uptake in skeletal muscle (Bakshi et al., 2018; Takahashi et al., 2020). Mitochondrial creatine kinase 2 (*CKMT2*) has been shown to be an important protein related to energy metabolism during muscle development in tissue in swine (Voillet et al., 2018), bovine (Cassar-Malek et al., 2017), chicken (Bottje et al., 2017), and other species. Multiple members of the ankyrin repeat and suppressor (*ASB*) family of cytokine signaling cassettes encoding E3 ubiquitination ligases are thought to be important genes during skeletal muscle growth and development (Ehrlich et al., 2020). It has been reported that ASB4 can promote vascular differentiation and myogenesis in mice by degrading protein ID2 (DNA-binding inhibitor 2), a protein that negatively regulates myogenesis and vascular differentiation (Townley-Tilson et al., 2014). *ASB15* has been shown to be involved in muscle cell development by promoting protein synthesis and regulating muscle cell differentiation (McDaneld et al., 2006). Also, multiple SNP loci of *ASB15* were significantly associated with chicken muscle-related carcass traits (Wang et al., 2015; Wang et al., 2017).

Ribosomal proteins are the main components involved in the composition of ribosomes. The ribosomal proteins are composed of the large subunit ribosomal protein (RPL) and the small subunit ribosomal protein (Wool, 1979). Many studies have shown that ribosomal proteins are involved in cell proliferation (Lindström and Zhang, 2008; Li et al., 2012), cell differentiation (Schneider et al., 2016), apoptosis (Khanna et al., 2003), regulation of cell development (Komili et al., 2007; Zhang et al., 2013), malignant transformation of normal cells (Challagundla et al., 2011; Pourdehnad et al., 2013) and extraribosomal functions (Warner and McIntosh, 2009). Due to the large number of ribosomal protein genes and limited current research methods, the expression patterns of ribosomal protein genes are still unclear (Uprety et al., 2018; Derenzini et al., 2019). Previous studies have demonstrated that reduced levels of ribosomal protein genes in organisms can adversely affect growth, development, and physiological functions. Most ribosomal protein genes exhibit specific expression patterns at specific developmental stages or specific tissues (Bortoluzzi et al., 2001), and haploinsufficiency of individual ribosomal proteins can lead to non-lethal phenotypic malformations and abnormal translation efficiency (Coelho et al., 2005; Marygold et al., 2005). There are 80 different ribosomal proteins in eukaryotic cells, among them, the ribosomal protein L3-like (RPL3L) is a member of the large subunit ribosomal protein. *RPL3L* was found to be a muscle-specifically expressed protein in humans and mice. Previous studies have shown that it was specific highly expressed in human muscle (Gupta and Warner, 2014) and a missense mutation in the human *RPL3L* gene could cause neonatal dilated cardiomyopathy (Ganapathi et al., 2020). The expression of *RPL3L* could significantly inhibit myotube formation in mouse myoblast C2C12 cells (Chaillou et al., 2016), and its knockdown in mice increases muscle fiber length (Kao et al., 2021). However, whether it plays a role in the growth and development of chicken skeletal muscle is still unknown. From the results of this study, we found that *RPL3L* has a certain interaction with several genes known to affect the growth and development of chicken skeletal muscle, such as *CKMT2*, *ASB4*, and *ASB15*. In this study, *RPL3L* was highly expressed in the proliferation and differentiation stages of primary myoblasts, respectively. It was found that *RPL3L* could inhibit the transcription of *ASB4* and *ASB15* genes in the cell proliferation stage but promote the transcription of them in the cell differentiation stage. Hence, we speculated that the chicken *RPL3L* may regulate muscle growth and development by affecting the expression of *ASB4* or *ASB15*.

The number of skeletal muscle fibers is mainly determined during the embryonic stage, and the increase in meat production is mainly due to the increase in muscle cell volume (Egner et al., 2016). Under the regulation of extracellular factors, neonatal skeletal muscle is mainly generated by the activation, proliferation, and differentiation of satellite cells (Morgan and Partridge., 2003; Dumont et al., 2015). Our study found that the *RPL3L* gene was significantly highly expressed in chicken skeletal muscle, and its expression was significantly increased after birth, which was consistent with the results obtained by previous studies of *RPL3L* in mammals. By studying its role in muscle satellite cells and myoblasts, it was found that *RPL3L* can significantly promote cell proliferation and inhibit cell differentiation. This result suggested that the molecular function of chicken *RPL3L* in skeletal muscle was the same as that of mammals. It shows that *RPL3L* was indeed a negative regulator gene involved in muscle growth and development. However, the specific signaling pathways through which this gene regulates muscle growth and development remain to be further explored.

As a new generation of molecular genetic markers, indel plays an important role in animal genetics and breeding research (Wang et al., 2013; Zang et al., 2016; Li et al., 2017). Previous studies have been applied to poultry genetics and breeding by mining effective indels of different muscle growth and development-related genes (Ren et al., 2020). We found an interesting indel of 42 bp by screening the genetic sequences of the chicken *RPL3L* gene in different breeds, and its DD genotype has a significant mutation rate in large chicken breeds. We speculated that it may be somewhat associated with weight-related traits in chickens. Therefore, using the F2 resource population of Xinghua chicken crossing with WRR chicken to conduct an association analysis of multiple economic traits and genotypes with different mutation sites, it was found that the DD genotype had significantly higher performance in weight-related traits, which may be an important weight-related molecular genetic marker. In this mutated region, we identified a loss of at least eight important transcription factor binding sites, which may contribute to the decreased transcriptional activity of *RPL3L* during transcription in individuals with the DD genotype. Lower transcriptional activity increased muscle mass production due to the negative regulatory role of *RPL3L* during muscle growth and development. The transcription factor c-Myc has previously been reported to inhibit the differentiation of primary myoblasts (Luo et al., 2019). It is speculated that individuals with the DD genotype have stronger muscle growth and development ability due to the failure of c-Mycbinding because of base deletion, thereby reducing the effect of c-Myc on inhibiting the differentiation of primary myoblasts.

## Conclusion

It was found from multiple datasets that *RPL3L*, *FBP2*, *ASB4*, *ASB15*, *CKMT2*, *PGAM1*, *YIPF7*, *PFKM*, and *LDHA* may be key genes affecting chicken muscle development. Among them, 42 bp indel in the *RPL3L* gene may be one of the molecular markers of chicken weight-related traits. In addition, the *RPL3L* could promote chicken myoblast proliferation and inhibit chicken myoblast differentiation to participate in chicken muscle development.

## Data Availability

The datasets presented in this study can be found in online repositories. The names of the repository/repositories and accession number(s) can be found in the article/Supplementary Material.

## Abbreviations

DD, deletion/deletion mutation; DEG, differentially expressed genes; GO, Gene Ontology; II, insertion/insertion mutation; Indel, insertion/deletion mutation; KEGG, Kyoto Encyclopedia of Genes and Genomes; RPL3L, ribosomal protein large 3-like; WRR, White Recessive Rock chickens.
