# Supplementary material for: Mining of chicken muscle growth genes and the function of important candidate gene RPL3L in muscle development
Source: Front Physiol. 2022 Nov 3;13:1033075. doi: 10.3389/fphys.2022.1033075 (PMC9669902; doi:10.3389/fphys.2022.1033075)
Supplement: Supplementary file 1 [file DataSheet1.docx]

Supplementary Figures and Tables


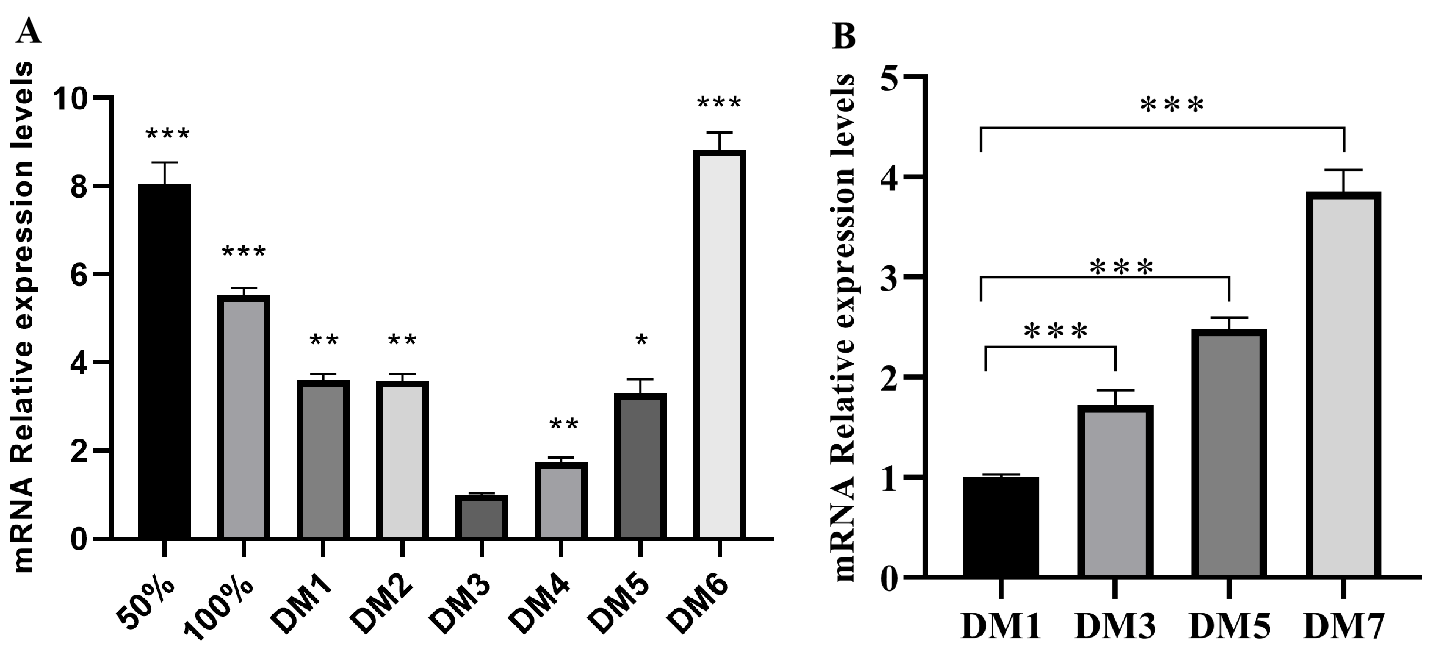


Supplementary Figure 1. The expression level of RPL3L in chicken primary myoblasts and satellite cells. (A) The expression level of RPL3L during the proliferation and differentiation of chicken primary myoblasts; (B) The expression level of RPL3L during the differentiation of chicken satellite cells. 50% and 100%, The density of cells after proliferation; DM1-DM7, cells cultured in differentiation medium for one to seven days.


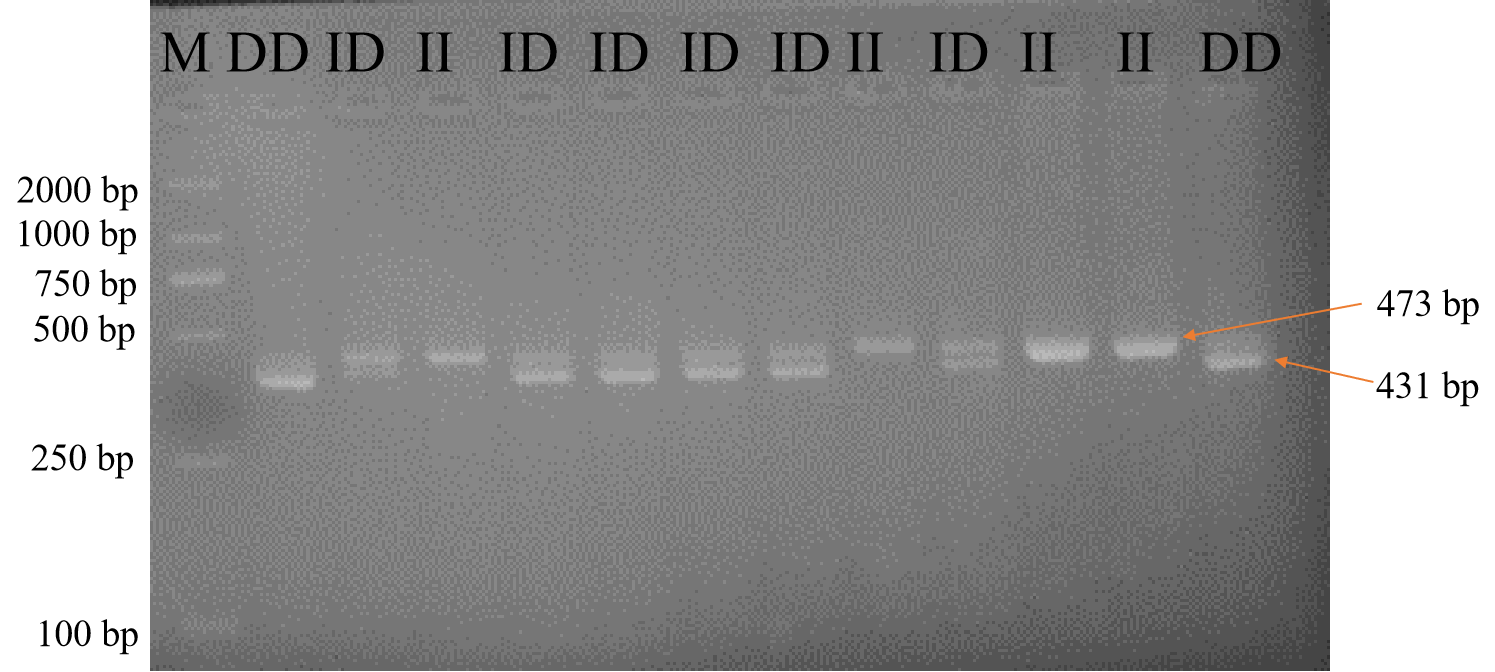


**Supplementary Figure 2.** Electrophoresis results of *RPL3L* 42 bp indel genotyping. M, maker; DD, deletion/deletion type, one band with 431 bp in size; ID, indel type, two bands with different size; II, insertion/insertion type, one band with 473 bp in size.


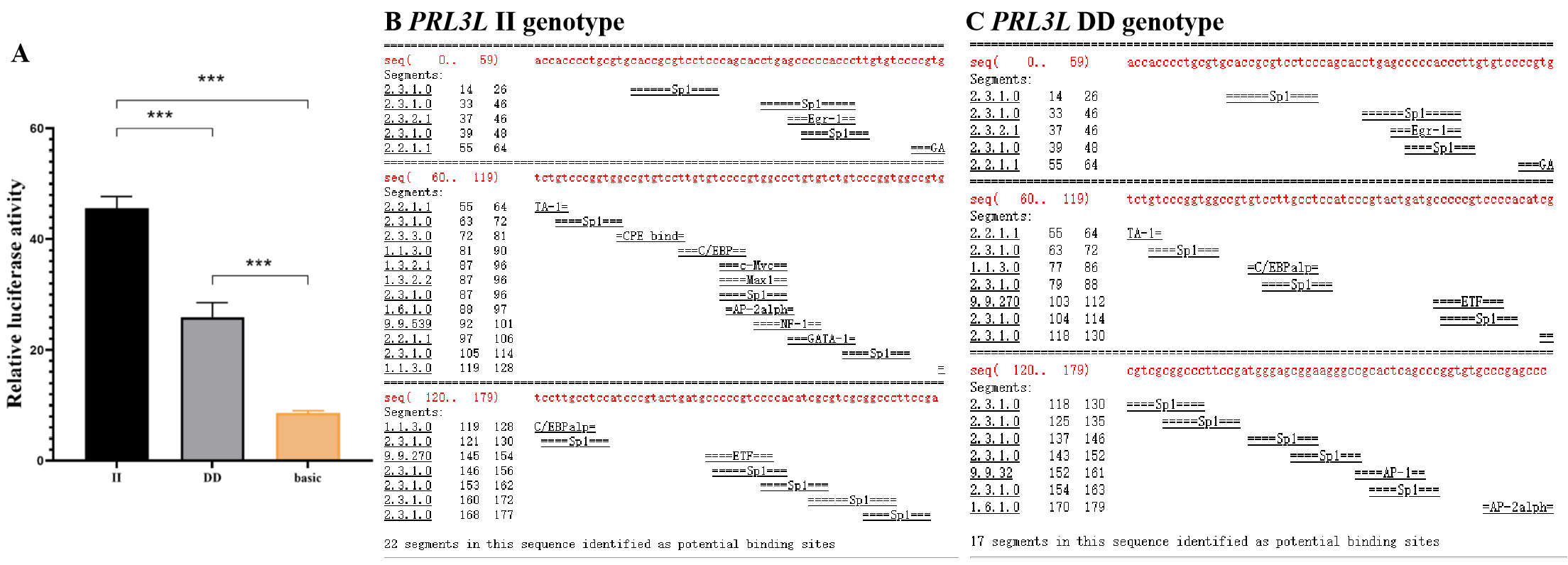


Supplementary Figure 3. Transcriptional activity and transcription factor binding site prediction of different genotypes of RPL3L 42 bp indel. (A) Transcriptional activity of RPL3L 42 bp indel in different genotypes. (B) Transcription factors binding site prediction of wild-type (II) in the 42 bp indel region of RPL3L; (C) Transcription factors binding site prediction of deletion mutant (DD) in the 42 bp indel region of RPL3L.

**Table S1 |** The information of RNAseq samples used in this study.

| **Dataset number** | **Database** | **Number of samples** | **Chicke breed** | **Sample introduction** | **References** |
| --- | --- | --- | --- | --- | --- |
| PRJCA001556 | NGDC | 11 | Shouguang Chicken | The breast muscle tissues of E12, E17, D1 and 8W | Liu, et al., 2019 |
| PRJCA001192 | NGDC | 12 | Jingxin Yellow Chicken | The breast muscle tissues of E12, E17, D1 and 8W | Xing, et al., 2020 |
| GSE162148 | GEO | 18 | Tibetan chicken | Skeletal muscle, liver, spleen, lung, kidney, abdominal fat | Jin, et al., 2021 |
| PRJNA665193 | ENA | 16 | White Leghorns | Skeletal muscle, liver, spleen, lung, abdominal fat, cerebellum, cerebral cortex, hypothalamus | Kern, et al., 2021 |

Note: E, embryo age; D, day age; W, week age; NGDC, the National Genomics Data Center, China National Center for Bioinformation; GEO, the Gene Expression Omnibus database; ENA, the European Nucleotide Archive database.

**Table S2 |** The primers used in this study.

| **Name** | **Sequence（5’-3’）** | | **Tm (℃)** | **Amplicon size** | **Application** |
| --- | --- | --- | --- | --- | --- |
| RPL3L | F | GCTCACTCTGCGGAAGTCCCT | 63 | 40 | qRT-PCR |
|  | R | AATGCACGCTTCTCCTGAGCTG |  |  |  |
| MyHC | F | CTCCTCACGCTTTGGTAA | 60 |  |  |
|  | R | TGATAGTCGTATGGGTTGGT |  |  |  |
| MyoMaker | F | TGGGTGTCCCTGATGGC | 60 |  |  |
|  | R | CCCGATGGGTCCTGAGTAG |  |  |  |
| MyoD | F | CAACGCCATCCGCTACATCG | 60 |  |  |
|  | R | TTTGGGTCATTTGGTGATTCCGT |  |  |  |
| MyoG | F | CGGAGGCTGAAGAAGGTGAA | 60 |  |  |
|  | R | CGGTCCTCTGCCTGGTCAT |  |  |  |
| Mef2C | F | ATCTCTCCCTGCCTTCTA | 60 |  |  |
|  | R | GTGATCTTCTCGGTCGCT |  |  |  |
| Myf5 | F | TTGAGGGAACAGGTGGAGAACTAC | 60 |  |  |
|  | R | GAGAGAGGCGGTCCACGATG |  |  |  |
| β-actin | F | GATATTGCTGCGCTCGTTG | 60 |  |  |
|  | R | TTCAGGGTCAGGATACCTCTTT |  |  |  |
| PAX7 | F | CAGCAACCGACGAGCAAGAT | 60 |  |  |
|  | R | GGTTGGGTAGGTGGAGTCT |  |  |  |
| ASB4 | F | GACTTCAAATCCCCGCTCCA | 60 |  |  |
|  | R | TTAACGACCACCTCCACAGC |  |  |  |
| ASB15 | F | GCTGGTGCAAATCCAAGCAA | 60 |  |  |
|  | R | AGGCATACTGGATGGCACTG |  |  |  |
| CKMT2 | F | ATGGGTCGATCAGAGGTGGA | 60 |  |  |
|  | R | CCAAACTGTGGCAATGGTGG |  |  |  |
| RPL3L-INDEL | F | CTGCCCAACCCGGTCACCT | 63 | 35 | Clone |
|  | R | CCCTCCAAGCACTCGCTCACGTC |  |  |  |

Note: F, forward primer; R, reverse primer; qRT-PCR, quantitative real-time PCR.

**Table S3 |** Clean reads data quality detection of four data sets used in this study.

| Data set | Sample | Reads (M) | Data volume (Gb) | Q20 (%) | Q30 (%) |
| --- | --- | --- | --- | --- | --- |
| PRJCA001556 | E12_1 | 71.89 | 10.79 | 98.16 | 94.67 |
|  | E12_2 | 68.37 | 10.25 | 98.14 | 94.61 |
|  | E12_3 | 70.26 | 10.51 | 97.50 | 93.35 |
|  | E17_1 | 73.32 | 10.98 | 98.24 | 94.81 |
|  | E17_2 | 64.94 | 9.75 | 98.06 | 94.42 |
|  | D1_1 | 71.93 | 10.76 | 98.12 | 94.55 |
|  | D1_2 | 82.52 | 12.16 | 98.78 | 95.99 |
|  | D1_3 | 72.35 | 10.84 | 98.13 | 94.56 |
|  | D56_1 | 84.52 | 12.68 | 98.18 | 94.96 |
|  | D56_2 | 85.71 | 12.86 | 97.97 | 94.47 |
|  | D56_3 | 94.20 | 14.14 | 98.06 | 94.65 |
| PRJCA001192 | E12B07 | 56.33 | 6.86 | 94.63 | 88.84 |
|  | E12B14 | 61.80 | 7.54 | 94.89 | 89.38 |
|  | E12B19 | 68.12 | 8.30 | 94.98 | 89.52 |
|  | E17B07 | 60.99 | 7.43 | 94.59 | 88.73 |
|  | E17B14 | 62.60 | 7.65 | 94.95 | 89.45 |
|  | E17B19 | 69.23 | 8.45 | 94.71 | 88.98 |
|  | D1B07 | 63.54 | 7.80 | 95.51 | 90.43 |
|  | D1B14 | 65.84 | 8.07 | 95.49 | 90.40 |
|  | D1B19 | 59.87 | 7.34 | 95.50 | 90.41 |
|  | D56B07 | 50.55 | 6.19 | 95.11 | 89.73 |
|  | D56B14 | 50.07 | 6.13 | 95.65 | 90.82 |
|  | D56B19 | 62.22 | 7.62 | 95.90 | 91.31 |
| GSE162148 | Chicken3-Adipose | 78.26 | 11.67 | 97.97 | 94.30 |
|  | Chicken3-Spleen | 105.84 | 15.80 | 97.30 | 93.07 |
|  | Chicken3-Lung | 93.00 | 13.89 | 97.63 | 93.80 |
|  | Chicken3-Liver | 86.68 | 12.96 | 98.19 | 95.03 |
|  | Chicken3-skeletal-muscle | 72.90 | 10.90 | 97.91 | 94.24 |
|  | Chicken3-Kidney | 80.54 | 12.03 | 97.61 | 93.73 |
|  | Chicken2-Adipose | 102.90 | 15.36 | 97.87 | 94.02 |
|  | Chicken2-Spleen | 80.90 | 12.08 | 97.33 | 93.14 |
|  | Chicken2-Lung | 102.86 | 15.37 | 97.77 | 94.13 |
|  | Chicken2-Liver | 113.27 | 16.94 | 98.27 | 95.21 |
|  | Chicken2-skeletal-muscle | 79.03 | 11.78 | 96.92 | 91.84 |
|  | Chicken2-Kidney | 100.15 | 14.97 | 97.74 | 94.02 |
|  | Chicken1-Adipose | 98.28 | 14.65 | 97.47 | 93.13 |
|  | Chicken1-Spleen | 102.88 | 15.36 | 97.34 | 93.17 |
|  | Chicken1-Lung | 93.63 | 13.96 | 97.58 | 93.68 |
|  | Chicken1-Liver | 96.82 | 14.48 | 98.14 | 94.90 |
|  | Chicken1-skeletal-muscle | 79.17 | 11.83 | 97.84 | 94.16 |
|  | Chicken1-Kidney | 127.53 | 19.05 | 97.66 | 93.86 |
| PRJNA665193 | Adipose1 | 98.91 | 9.65 | 98.83 | 96.10 |
|  | Adipose2 | 136.89 | 13.35 | 98.85 | 96.35 |
|  | Cerebellum1 | 146.61 | 14.43 | 98.84 | 96.34 |
|  | Cerebellum2 | 140.18 | 13.42 | 98.89 | 96.34 |
|  | Cortex1 | 151.39 | 14.48 | 98.85 | 96.30 |
|  | Cortex2 | 135.09 | 13.17 | 98.87 | 96.39 |
|  | Hypothalamus1 | 143.60 | 14.11 | 98.77 | 96.31 |
|  | Hypothalamus2 | 143.16 | 13.85 | 98.90 | 96.48 |
|  | Liver1 | 110.65 | 10.94 | 98.83 | 96.14 |
|  | Liver2 | 157.36 | 15.59 | 98.85 | 96.30 |
|  | Lung1 | 112.51 | 11.09 | 98.33 | 94.86 |
|  | Lung2 | 119.71 | 11.81 | 98.79 | 96.14 |
|  | Skeletal muscle1 | 147.00 | 14.42 | 98.78 | 95.87 |
|  | Skeletal muscle2 | 146.50 | 14.45 | 98.87 | 96.16 |
|  | Spleen1 | 120.32 | 11.87 | 98.64 | 95.70 |
|  | Spleen2 | 116.47 | 11.31 | 98.86 | 96.20 |

Note: E, embryonic age; D, day age; Q20 and Q30 indicating the probability of base misidentification in sequencing was 1% and 0.1%, respectively.

**Table S4 |** The 79 postnatal muscle-specific highly expressed genes.

| **DEGs** | **DEGs** | **DEGs** | **DEGs** |
| --- | --- | --- | --- |
| ABCB6 | CKMT2 | JPH1 | PFKM |
| ABCF3 | CLCN1 | KBTBD12 | PGAM1 |
| ACTR3B | COQ9 | KLHL38 | PGM1 |
| ACYP2 | CUL4A | LDHA | PHKA1 |
| AGL | CYCS | LOC101748756 | PHKG1 |
| AK1 | DUPD1 | LOC423731 | PKLR |
| ALPK3 | DUSP13 | LOC431653 | PPP1R3C |
| ANKRD1 | EEF1A2 | LRRC30 | RILPL1 |
| ANO8 | ENSGALG00000034083 | METTL21EP | RPL3L |
| ASB11 | ENSGALG00000049432 | MHM2 | SDHA |
| ASB15 | ENSGALG00000051225 | MLF1 | SLC25A12 |
| ASB4 | ENSGALG00000053585 | MRPL34 | SLC25A4 |
| ATG9B | ENSGALG00000054604 | MUL1L | SLC28A2 |
| ATP2A3 | FASTK | MYH1F | TMOD4 |
| BEST3 | FBP2 | MYOZ3 | TOM1 |
| BTBD2 | FBXL4 | NMRK2 | TUBA8A |
| C25H1ORF43 | FBXO40 | OPTN | USP13 |
| CAC1S | FYCO1 | PCOLCE2 | VDAC1 |
| CAND2 | GAPDH | PEBP4 | YIPF7 |
| CDC42EP3 | GYS1 | PFKFB4 |  |

Note: DEGs, differentially expressed genes.
